# Polyamine Resistance Is Increased by Mutations in a Nitrate Transporter Gene *NRT1.3* (*AtNPF6.4*) in *Arabidopsis thaliana*

**DOI:** 10.3389/fpls.2016.00834

**Published:** 2016-06-13

**Authors:** Wurina Tong, Akihiro Imai, Ryo Tabata, Shuji Shigenobu, Katsushi Yamaguchi, Masashi Yamada, Mitsuyasu Hasebe, Shinichiro Sawa, Hiroyasu Motose, Taku Takahashi

**Affiliations:** ^1^Graduate School of Natural Science and Technology, Okayama UniversityOkayama, Japan; ^2^National Institute for Basic BiologyOkazaki, Japan; ^3^Graduate School of Science and Technology, Kumamoto UniversityKumamoto, Japan; ^4^Department of Biology, Duke UniversityDurham, NC, USA

**Keywords:** *Arabidopsis*, nitrate transporter, NRT1/PTR family, parenchymal tissue, polyamines

## Abstract

Polyamines are small basic compounds present in all living organisms and act in a variety of biological processes. However, the mechanism of polyamine sensing, signaling and response in relation to other metabolic pathways remains to be fully addressed in plant cells. As one approach, we isolated *Arabidopsis* mutants that show increased resistance to spermine in terms of chlorosis. We show here that two of the mutants have a point mutation in a nitrate transporter gene of the *NRT1/PTR* family (NPF), *NRT1.3* (*AtNPF6.4*). These mutants also exhibit increased resistance to putrescine and spermidine while loss-of-function mutants of the two closest homologs of *NRT1.3*, root-specific *NRT1.1* (*AtNPF6.3*) and petiole-specific *NRT1.4* (*AtNPF6.2*), were shown to have a normal sensitivity to polyamines. When the *GUS* reporter gene was expressed under the control of the *NRT1.3* promoter, GUS staining was observed in leaf mesophyll cells and stem cortex cells but not in the epidermis, suggesting that *NRT1.3* specifically functions in parenchymal tissues. We further found that the aerial part of the mutant seedling has normal levels of polyamines but shows reduced uptake of norspermidine compared with the wild type. These results suggest that polyamine transport or metabolism is associated with nitrate transport in the parenchymal tissue of the shoot.

## Introduction

Polyamines such as putrescine and spermidine are small basic compounds present in all living organisms and play a role in a variety of biological processes including DNA replication, protein synthesis, and ion channel modulation (Igarashi and Kashiwagi, 2010b; Pegg and Casero, 2011). The cellular content of polyamines is highly regulated by biosynthesis, oxidative degradation, transport, and, in plants, conjugation to hydroxycinnamic acids. Both in animals and plants, most of the mRNAs of *S*-adenosylmethionine decarboxylase (AdoMetDC), which is a key enzyme providing the aminopropyl group for the synthesis of spermidine and spermine, contain small upstream open reading frames (uORFs) that mediate polyamine-dependent repression of the main ORF translation (Raney et al., 2002; Hanfrey et al., 2005). Plants and some bacteria have a structural isomer of spermine, thermospermine (Takano et al., 2012). We have found that expression of *ACAULIS5* (*ACL5*), which encodes thermospermine synthase in *Arabidopsis*, is under negative feedback control by thermospermine (Kakehi et al., 2008; Tong et al., 2014). In animals and fungi, degradation of ornithine decarboxylase (ODC), which catalyzes the conversion of ornithine into putrescine, a rate-limiting step in polyamine biosynthesis, is directed by ODC antizyme (OAZ) whose synthesis is induced by higher cellular polyamine levels (Matsufuji et al., 1995; Ivanov et al., 2000).

As for transport systems, antiporters of putrescine-ornithine and cadaverine-lysine as well as putrescine-specific and spermidine-preferential uptake carriers have been characterized in *Escherichia coli* (Kashiwagi and Igarashi, 2011). In yeast, GAP1 catalyzes the uptake of putrescine and spermidine together with the uptake of amino acids (Uemura et al., 2005). AGP2 acts as a high-affinity amino acid permease and selectively catalyzes the uptake of spermidine (Aouida et al., 2005). Three additional proteins, DUR3, SAM3, and tonoplast-localized UGA4 also function in polyamine uptake, while five excretion proteins, TPO1 to TPO5, have been identified (Igarashi and Kashiwagi, 2010a). In human, while multiple systems for uptake of polyamines have been identified, the organic cation transporter2 (OCT2) has been shown to function as a common uptake carrier (Higashi et al., 2014). In plants, earlier studies using petals of *Saintpaulia ionantha* revealed that putrescine uptake is dependent on the external pH (Bagni and Pistocchi, 1985). In carrot cell cultures, the uptake of putrescine and spermidine is remarkably rapid, reaching a maximum within only 1 min (Pistocchi et al., 1987; Antognoni et al., 1993). A genetic study of an *Arabidopsis* wild-type accession that is resistant to paraquat identified RMV1 as a polyamine uptake transporter (Fujita et al., 2012). In rice, OsPUT1 to OsPUT3 have been identified as spermidine-preferential transporters. AtPUT1 to AtPUT3 are the orthologous proteins in *Arabidopsis* and function as high affinity spermidine uptake transporters (Mulangi et al., 2012), among which AtPUT3 is identical to RMV1 (Fujita and Shinozaki, 2014).

Degradation of spermidine, spermine, and thermospermine in plants is catalyzed by polyamine oxidases (PAOs). PAOs in peroxisomes or the cytoplasm mediate back-conversion reaction with an end product of hydrogen peroxide and probably 3-aminopropanal, while extracellular PAOs catalyze terminal catabolic reactions to produce 1,3-diaminopropane and hydrogen peroxide (Moschou et al., 2008; Tavladoraki et al., 2012). Putrescine is catalyzed by copper-containing amine oxidases (CuAOs) to 4-aminobutanal along with ammonia and hydrogen peroxide (Angelini et al., 2010). Polyamine-derived hydrogen peroxide plays a critical role in biotic and abiotic stress responses and also in triggering secondary wall deposition (Cona et al., 2006; Moschou et al., 2012; Moschou and Roubelakis-Angelakis, 2014).

While cellular polyamine levels are maintained by the above-described regulatory systems, they may also be interrelated with other metabolic pathways. In poplar cell cultures, spermidine and spermine levels positively correlate with most amino acids (Mattoo et al., 2010). Increased polyamine biosynthesis may result in increased assimilation of both nitrogen and carbon by the cells (Majumdar et al., 2016). On the other hand, exogenous polyamines cause a rapid production of nitric oxide (NO) in *Arabidopsis* (Tun et al., 2006). A genetic screen of *Arabidopsis* mutants with increased tolerance to norspermidine identified a dominant mutant, *par1-1D*, with increased expression of *QSO2* (Alejandro et al., 2007). *QSO2* encodes a quiescin-sulfhydryl oxidase and may activate K^+^ eﬄux systems involved in xylem loading in roots thereby reducing the accumulation of toxic cations such as Na^+^ and polyamines. We are interested in how plant cells perceive and respond to external polyamines. To gain insight into sensing, signaling, and responsive mechanisms to polyamines, we isolated *Arabidopsis* mutants that show increased resistance to spermine. Among them, two mutants were found to be alleles of *NRT1.3* encoding a member of the low-affinity nitrate transporter family.

## Materials and Methods

### Chemicals

The hydrochloride salts of putrescine, spermidine, spermine, and norspermidine were purchased from Sigma (MO, USA). Murashige and Skoog (MS) salts were purchased from Wako (Osaka, Japan).

### Plant Materials

*Arabidopsis thaliana* accession Columbia-0 (Col-0) was used as the wide type. A T-DNA insertion allele of *NRT1.3* (*AtNPF6.4*), which was obtained from the SALK collection (SALK_001553) and named here *sper3-3*, and a deletion allele of *NRT1.1* (*AtNPF6.3*), *chl1-5* (Tsay et al., 1993), were obtained from the *Arabidopsis* Biological Resource Center (ABRC) at Ohio State University (OH, USA). A T-DNA insertion allele of *NRT1.4* (*AtNPF6.2*), *nrt1.4-2* (Chiu et al., 2004), was a gift from Yi-Fang Tsay (Academia Sinica, Taiwan). A T-DNA insertion allele of *NRT1.2* (*AtNPF4.6*), *ait1-1* (SALK_146143; Kanno et al., 2012), and a paraquat-resistant accession Est-1 (Fujita et al., 2012) were also obtained from ABRC. *spms-1* has been previously described (Imai et al., 2004). The wild-type accession Landsberg *erecta* (L*er*) was used for mapping experiments.

### Mutant Isolation, Mapping, and Next Generation Sequencing

For screening of mutants resistant to polyamines, 49,000 M2 seeds of ethyl methanesulfonate (EMS)-mutagenized Col-0 were sown on MS agar plates containing 3 mM spermine and 28 resistant plant lines were selected. Among them, three lines that reproducibly showed increased resistance to spermine in the progeny were named *spermine resistant1* (*sper1*), *sper2*, and *sper3*, respectively.

For chromosomal mapping of the mutant loci, F2 seeds from the cross between the mutants and L*er* were sown on MS agar plates containing 3 mM spermine and the DNA was extracted from each individual that was resistant to spermine. Totally ca. 80–100 seedlings were selected for each mutant. PCR-based mapping was performed using simple sequence length polymorphism (SSLP) markers (Bell and Ecker, 1994) and cleaved amplified polymorphic sequence (CAPS) markers (Konieczny and Ausubel, 1993). Additional markers were designed according to the TAIR database^1^ and the primers used are shown in Supplementary Table S1.

Genome DNA sequences of the mutants were determined by next-generation sequencing with the SOLiD platform. The multiplex libraries were constructed using the SOLiD barcoding and sequenced on a single SOLiD slide (Tabata et al., 2013). The resulting sequence reads were aligned against the *A. thaliana* Col-0 genome reference TAIR9 (TAIR_chr_all.fas) using BioScope 1.3 software (Life Technologies) with default parameters. Single nucleotide polymorphisms (SNPs) were called using a diBayes SNP caller, a component of BioScope 1.3 and validated as described in Tabata et al. (2013).

### Plasmid Construction and Plant Transformation

To generate the *NRT1.3* promoter-*GUS* fusion gene, a 1.47-kb promoter region of *NRT1.3* was amplified from Col-0 genomic DNA by PCR with primers SPER3pro-F, 5′-CACAG ACTCT TGGTT TCTAG A-3′ and SPER3pro-R, 5′-GGATC CTGAG AGAAG AAAGC AGAG-3′ and the PCR product was cloned into the pGEM-T easy vector (Promega), excised by restriction digestion with *Xba*I and *Bam*HI, and cloned into the pBI101 Ti-plasmid vector (Clontech).

To construct the 35S promoter-driven *NRT1.3-GFP* fusion plasmid, a full-length cDNA of *NRT1.3* was amplified by PCR with primers SPER3-F-ATG, 5′-CACCA TGGTT CATGT GTCAT CATCT CATG-3′ and SPER3-R-dSTOP, 5′-AGGAA TGTCT TAAGC TCAAA TTCG-3′ and the PCR product was cloned into the pENTR/D-TOPO vector (Invitrogen). Then, the fragment was transferred to the C-terminal GFP fusion vector pGWB5 (Nakagawa et al., 2007).

The constructs were introduced into *Agrobacterium tumefaciens* C58C1 by electroporation. Transformation of *Arabidopsis* with the *GUS* construct was performed by the floral dip method (Harrison et al., 2006) using 0.05% Silwet L-77 in the dipping solution.

### Measurement of Chlorophyll

Chlorophyll was extracted from 50 mg of the aerial part of 10-day-old seedlings in 1 mL of N,N-dimethyl formamide at 4°C overnight in the dark and assayed as described (Porra et al., 1989).

### GUS Staining

Samples were prefixed for 20 min in ice-cold 90% acetone (v/v), vacuum infiltrated for 20 min with the GUS staining solution containing 50 mM sodium phosphate buffer pH7.2, 2 mM potassium ferricyanide, 2 mM potassium ferrocyanide, 0.1% Triton X-100 (v/v) and 1 mM X-glucuronide, and incubated at 37°C overnight (Jefferson et al., 1987). The stained samples were cleared with 70% ethanol and observed under a light microscope. For sectioning, GUS-stained tissues were dehydrated in a graded ethanol series and embedded in Technovit 7100 (Heraeus Kulzer, Wehrheim, Germany) according to the manufacturer’s instructions. Sections were cut to 10 μm.

### Transient Expression of GFP in Tobacco

*Agrobacterium* cells containing the *NRT1.3-GFP* construct were cultured in Luria broth (LB) with 50 μg/ml of kanamycin and hygromycin at 28°C overnight. *Agrobacterium* cells containing the p19 plasmid as a suppressor of silencing (Silhavy et al., 2002) were cultured in LB with 50 μg/ml kanamycin. Both cultures were centrifuged, dissolved in 10 ml infiltration buffer (10 mM MES, 10 mM MgCl_2_, 100 μM acetosyringone), and mixed together. The suspension was adjusted to a final OD_600_ of 0.8 for infiltration into leaves of 4-week-old *Nicotiana benthamiana.* At 2 days after infiltration, the GFP fluorescence signals were observed in the epidermal cells under confocal laser scanning microscopy (Eclipse C1, Nikon, Tokyo, Japan).

### HPLC Analysis

Cellular polyamine levels were measured by HPLC. Acid-soluble free polyamines were extracted with perchloric acid from the aerial part of 10-day-old seedlings and benzoylated as described previously (Tong et al., 2014). The samples were analyzed using a reverse phase HPLC system equipped with TSKgel ODS-80Ts column (Toso, Tokyo, Japan).

## Results

### *sper3-1* and *sper3-2* Are Resistant to Polyamines

**FIGURE 1:**
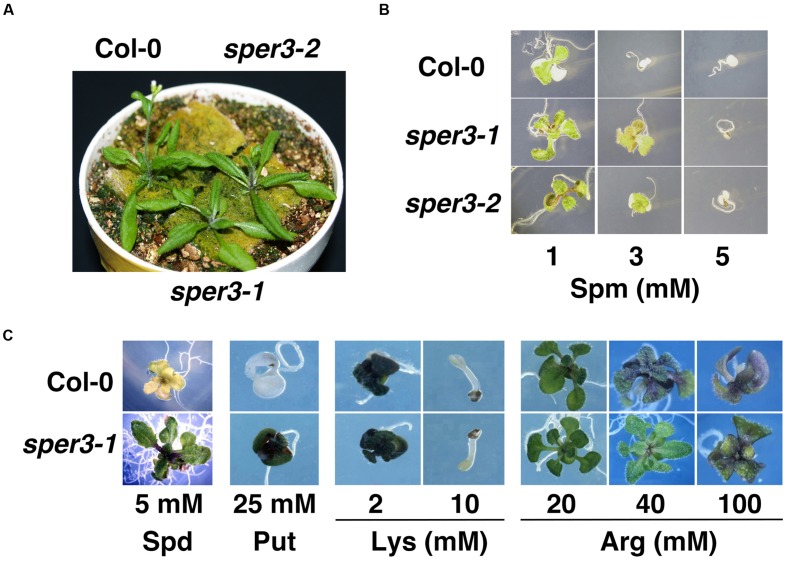
**Phenotypes of *sper3-1* and *sper3-2*. (A)** Gross morphology of 20-day-old wild-type (Col-0), *sper3-1* and *sper3-2* plants. **(B)** Phenotype of 10-day-old seedlings grown in MS agar plates with spermine (Spm). **(C)** Phenotype of 10-day-old seedlings grown in MS agar plates with spermidine (Spd), putrescine (Put), lysine (Lys), or arginine (Arg).

**FIGURE 2:**
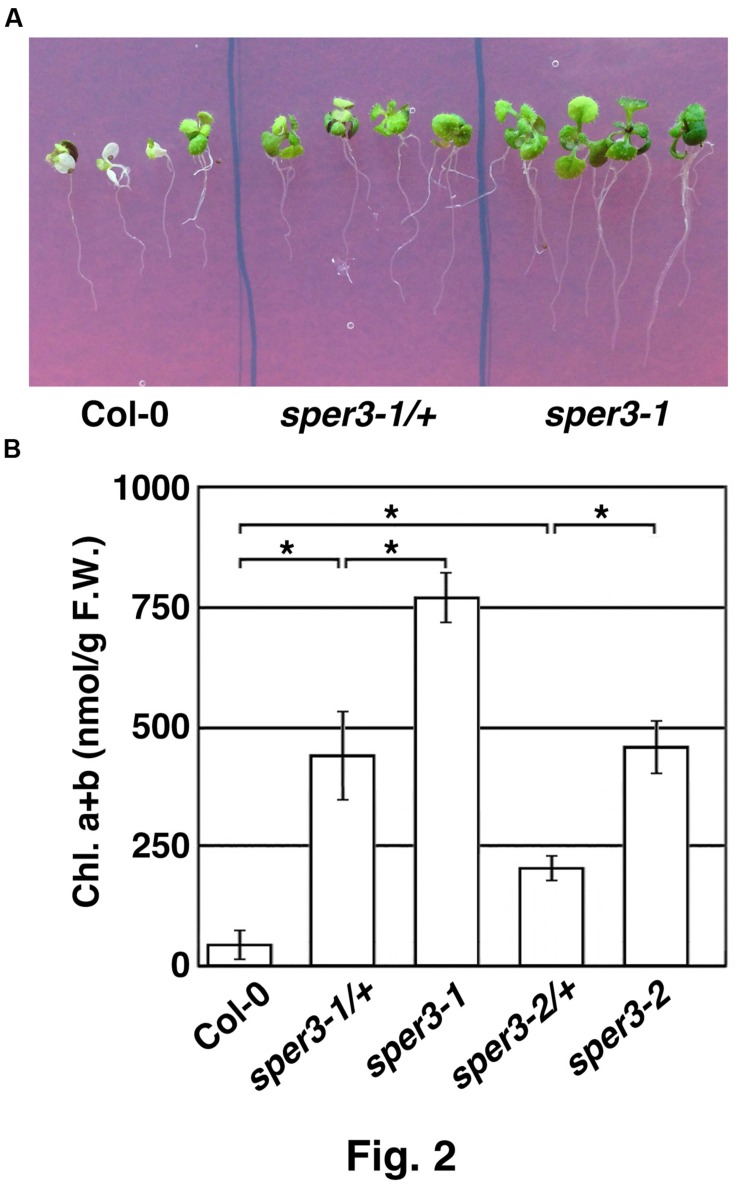
**Semi-dominant traits of *sper3-1* and *sper3-2*. (A)** Phenotype of 10-day-old seedlings grown in MS agar plates with 2 mM spermine. **(B)** Chlorophyll content of 10-day-old seedlings grown in MS agar plates with 2 mM spermine. Error bars represent the SE (*n* = 3). Asterisks indicate statistically significant difference between groups assessed by ANOVA with Tukey-Kramer HSD (*P* < 0.05).

We identified three mutants that showed increased resistance to 3 mM spermine in an M2 population derived from EMS-mutagenized seeds of the accession Col-0 of *A. thaliana* and tentatively named *sper1*, *sper2*, and *sper3*. Because rough mapping data showed that *sper1* is located on chromosome 4 while *sper2* and *sper3* are on chromosome 3 and, as shown below, *sper3* and *sper2* were found to be different alleles of the same gene, these alleles were designated *sper3-1* and *sper3-2*, respectively. Under normal growth conditions, *sper3-1* and *sper3-2* have no obvious morphological abnormalities compared with the wild type (**Figure 1A**). However, when grown on MS agar plates containing different concentrations of spermine, *sper3-1* and *sper3-2* seedlings retained green leaves at 3 mM spermine while wild-type seedlings showed chlorosis at 1 mM spermine (**Figure 1B**). These mutant seedlings were also resistant to 25 mM putrescine and 5 mM spermidine while they were no more resistant to basic amino acids, lysine and arginine, than wild-type seedlings (**Figure 1C** and not shown). F1 seedlings from a backcross of *sper3-1* to its wild-type parent Col-0 showed intermediate sensitivity to spermine in terms of the leaf development (**Figure 2A**) and the chlorophyll content (**Figure 2B**), indicating that *sper3-1* represents a semi-dominant allele. Similarly, the seedlings heterozygous for *sper3-2* showed intermediate sensitivity to spermine (**Figure 2B**), indicating that *sper3-2* also has a semi-dominant effect.

### *sper3-1* and *sper3-2* Are Alleles of *NRT1.3*

**FIGURE 3:**
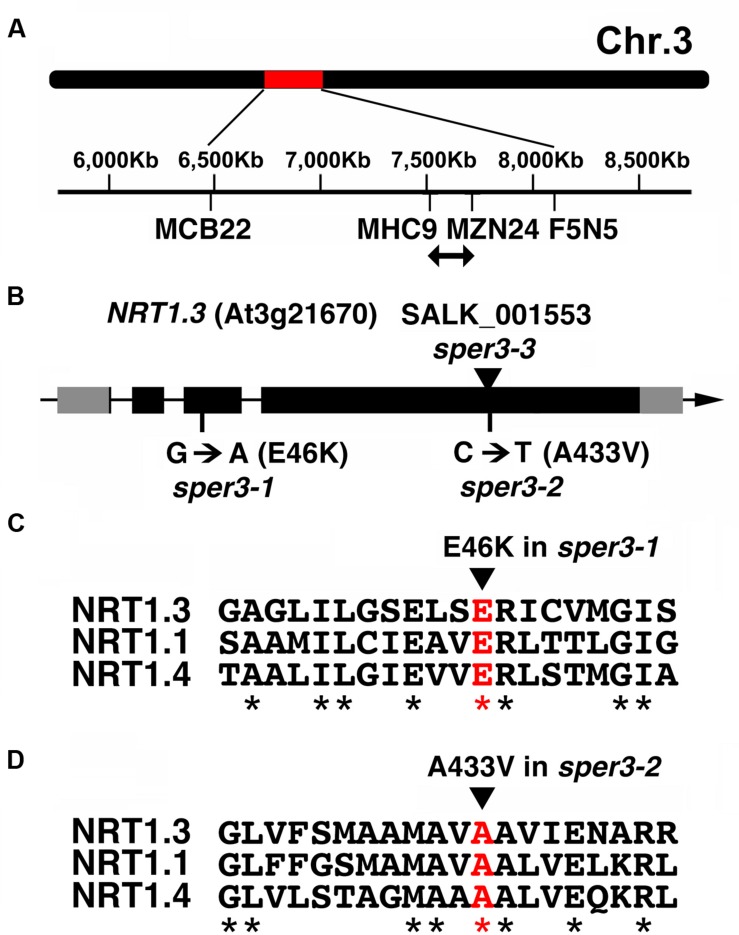
**Gene structure of *SPER3.* (A)** Chromosome location of *sper3* alleles. **(B)** Genomic structure of *NRT1.3* (*AtNPF6.4*, *SPER3*). Boxes indicate exons. Gray and black areas represent untranslated regions and protein coding regions, respectively. Base and amino acid substitutions in *sper3-1* and *sper3-2* are shown. The arrowhead indicates the position of T-DNA insertion in *sper3-3*. **(C,D)** Amino acid alignment of the region surrounding *sper3-1* **(C)** and *sper3-2* **(D)** mutated residues of NRT1.3 with corresponding regions of NRT1.1 (AtNPF6.3) and NRT1.4 (AtNPF6.2). Asterisks indicate conserved amino acids.

Fine mapping using F2 plants from a cross between *sper3-1* and the accession L*er* delimited the locus to a 200-kb region on chromosome 3 (**Figure 3A**). Next-generation sequencing data of this region of the *sper3-1* genomic DNA revealed a G-to-A point mutation in *NRT1.3* (*AtNPF6.4*; **Figure 3B**), which belongs to the NRT1/PTR low-affinity nitrate transporter family (Tsay et al., 2007; Supplementary Figure S1). The G-to-A base substitution in *sper3-1* changes glutamate 46, which is conserved in the family, to lysine (**Figure 3C**). On the other hand, sequencing of the *sper3-2* genomic DNA revealed a C-to-T base substitution that changes alanine 433 of *NRT1.3* (**Figure 3B**), which is also conserved in the family, to valine (**Figure 3D**).

**FIGURE 4:**
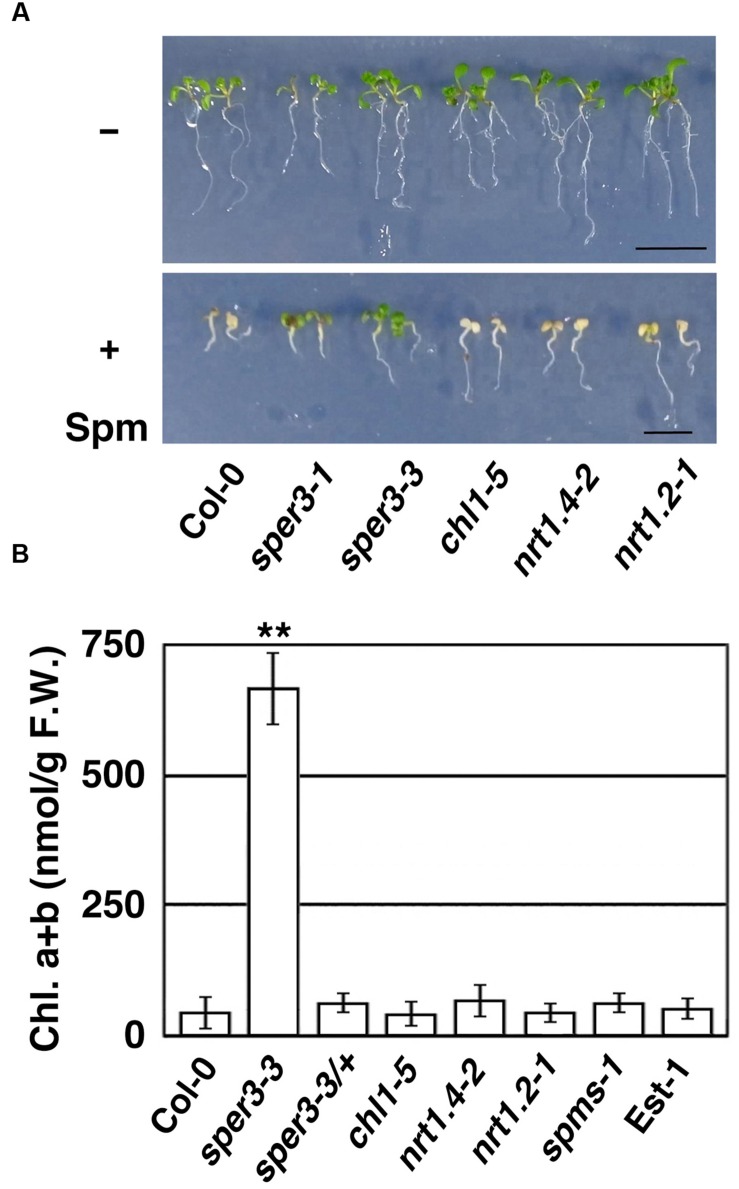
**Growth response of *sper3-3* and other knockout mutants of the *AtNPF6* subfamily to spermine. (A)** Phenotype of 7-day-old seedlings grown in MS agar plates with (+) or without (-) 2 mM spermine (Spm). *chl1-5*, *nrt1.4-2*, and *nrt1.2-1* (*ait1-1*) represent loss-of-function mutants of *AtNPF6.3*, *AtNPF6.2*, and *AtNPF4.6*, respectively. **(B)** Chlorophyll content of 7-day-old seedlings grown in MS agar plates with 2 mM spermine. Error bars represent the SE (*n* = 3). Asterisks indicate statistically significant difference between the wild-type and mutants (^∗∗^*P* < 0.01 Student’s *t*-test).

To confirm further that polyamine resistance is indeed caused by a mutation in *NRT1.3*, we examined whether a T-DNA insertion mutant of *NRT1.3* shows increased resistance to spermine. The results revealed that the homozygous knockout allele designated *sper3-3* (**Figure 3B**) had no detectable levels of full-length *NRT1.3* mRNA (Supplementary Figure S2) and clearly showed increased resistance to 3 mM spermine (**Figure 4A**). Unlike *sper3-1/*+ and *sper3-2/*+, heterozygous *sper3-3/*+ seedlings showed no increased resistance to spermine as shown in the chlorophyll content (**Figure 4B**), indicating that *sper3-3* is a recessive allele.

### *NRT1.3* Is Preferentially Expressed in Parenchymal Tissues

**FIGURE 5:**
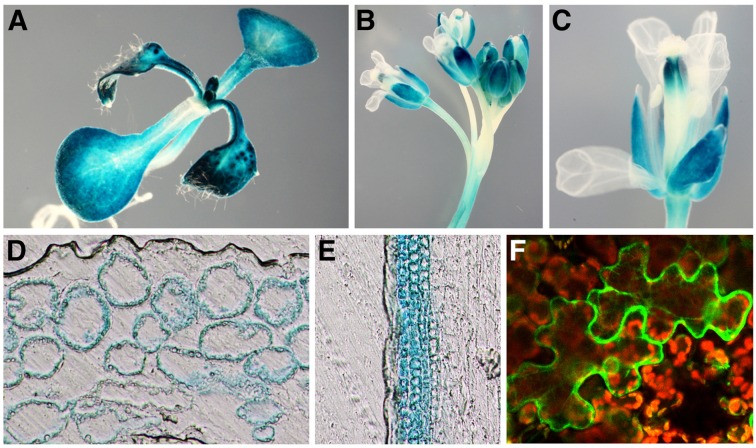
**Expression of the *AtNPF6.4* promoter-*GUS* gene and cellular localization of the AtNPF6.4-GFP fusion protein.** **(A–E)** GUS staining pattern of a transgenic *Arabidopsis* plant carrying the *AtNPF6.4* promoter-*GUS* fusion construct in 7-day-old seedlings **(A)**, floral shoots **(B)**, a single flower **(C)**, a leaf section **(D)**, and a stem section **(E)**. **(F)** GFP fluorescence in a tobacco leaf epidermal cells carrying the *AtNPF6.4-GFP* fusion construct.

*NRT1.3* expression was detected by RT-PCR in all organs examined with the highest expression in flowers (Supplementary Figure S3A). When wild-type seedlings grown for 7 days on MS agar plates were incubated for 2 or 24 h in MS solutions with 100 μM spermine, no significant changes in the level of the *NRT1.3* mRNA were detected (Supplementary Figure S3B). Although *NRT1.3* expression is induced by NO_3_^–^ in shoots (Okamoto et al., 2003), its tissue expression pattern has not been investigated in previous studies. Thus, we generated transgenic plants carrying the *NRT1.3* promoter-*GUS* fusion gene and examined the *GUS* expression in more than eight independent plant lines. GUS staining was detected in the hypocotyl, cotyledons, and leaves but not in the root of seedlings (**Figure 5A**). In adult plants, inflorescence stems, pedicels, and sepals of flowers were strongly stained (**Figures 5B,C**). Tissue sections revealed that *GUS* expression was restricted to mesophyll cells of leaves and cortical cells of stems but not in epidermal cells (**Figures 5D,E**), suggesting that *NRT1.3* specifically functions in parenchymal tissues.

To examine cellular localization of NRT1.3, an *NRT1.3-GFP* translational fusion gene was constructed and transiently expressed under the CaMV 35S promoter in tobacco leaves by agro-infiltration. The *NRT1.3-GFP* fluorescence was detected mainly along the plasma membrane (**Figure 5F**).

### Accumulation and Uptake of Polyamines in *sper3-1*

**FIGURE 6:**
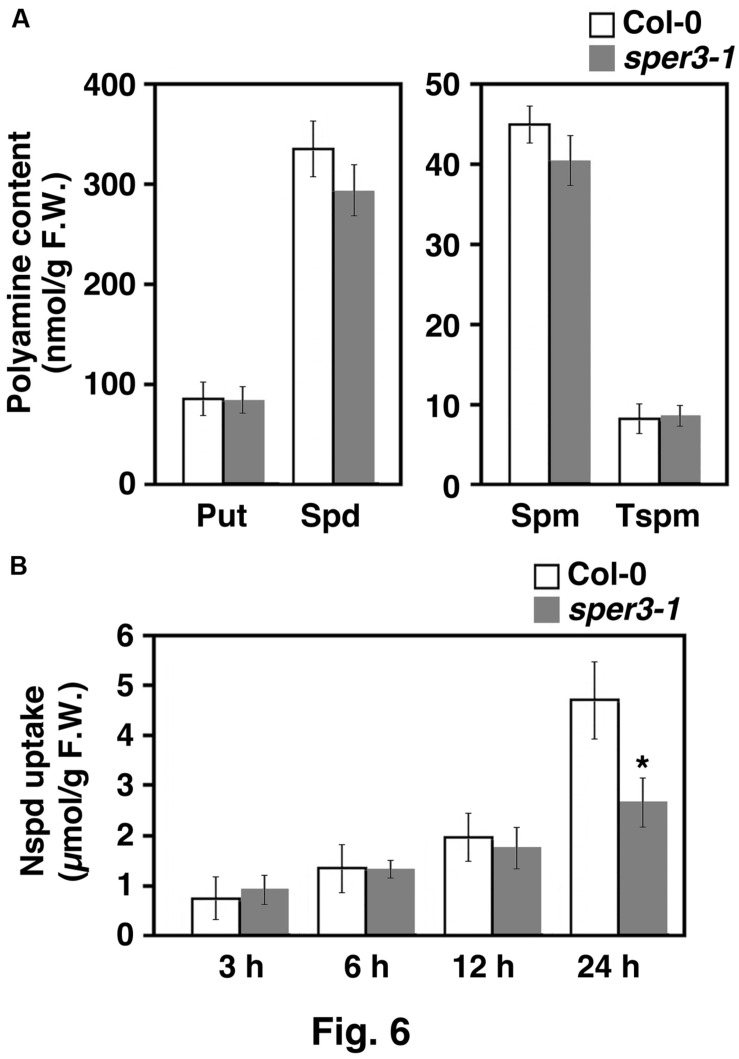
**Polyamine content of wild-type and *sper3-1* seedlings. (A)** Level of putrescine (Put), spermidine (Spd), spermine (Spm), and thermospermine (Tspm) in grown in MS agar plates. Polyamines were extracted and analyzed by HPLC as described in section “Materials and Methods.” **(B)** Level of norspermidine (Nspd) in above-ground parts of seedlings. Wild-type and *sper3-1* seedlings were grown for 7 days in MS agar plates, transferred to plates containing 100 μM norspermidine, and incubated for indicated hours. Error bars represent the SE (*n* = 3). An asterisk indicates statistically significant difference between wild-type and *sper3-1* (^∗^*P* < 0.05 Student’s *t*-test).

To examine whether or not *sper3-1* affects polyamine transport or metabolism, cellular polyamine levels in the aerial part of *sper3-1* seedlings were quantified by HPLC. The results revealed that the levels of all four polyamines, putrescine, spermidine, spermine, and thermospermine, were not affected by *sper3-1* (**Figure 6A**). We next investigated polyamine uptake activity in *sper3-1* by using norspermidine as a tracer because it is not present in wild-type plants. Seedlings grown for 7 days on MS agar plates were placed on the plate containing 100 μM norspermidine and the aerial part was subjected to polyamine extraction and HPLC. The results showed that the uptake level of norspermidine was initially indistinguishable between the wild type and *sper3-1* but significantly declined after 24 h incubation in *sper3-1* (**Figure 6B**).

### Mutants of *NRT1.1* and *NRT1.4* Show no Increased Resistance to Spermine

*NRT1.3* is classified into the same subfamily as root-specific *NRT1.1* (*AtNPF6.3*) and leaf petiole-specific *NRT1.4* (*AtNPF6.2*; Supplementary Figure S1; Wang et al., 2012). We thus examined whether these loss-of-function mutants and a mutant of vascular-specific *NRT1.2* (*AtNPF4.6*; Kanno et al., 2012) also show increased resistance to spermine or not. *chl1-5* is a deletion allele of *NRT1.1* which shows increased resistance to chlorate (Tsay et al., 1993), while *nrt1.4-2* and *ait1-1* represent T-DNA insertion mutants of *NRT1.4* and *NRT1.2*, respectively (Chiu et al., 2004; Kanno et al., 2012). When grown with 3 mM spermine, however, these mutant seedlings showed growth inhibition and chlorosis in a similar degree to wild-type seedlings (**Figure 4A**). Furthermore, neither *spms-1*, which is defective in the biosynthesis of spermine (Imai et al., 2004), nor the natural accession Est-1, which is defective in a plasma membrane-localized polyamine uptake transporter, RMV1/AtPUT3 (Fujita et al., 2012), showed increased resistance to spermine under our experimental condition (**Figure 4B**). We have also confirmed that, unlike the *RMV1/AtPUT3*-defective Est-1, which is resistant to paraquat, *sper3-1* showed normal sensitivity to paraquat (Supplementary Figure S4).

## Discussion

In this study, we found that alleles of *NRT1.3* encoding a member of the NRT1/PTR family of nitrate transport proteins are responsible for the mutants with increased resistance to polyamines. The NRT1/PTR family in *Arabidopsis* consists of 53 members and includes both nitrate and oligopeptide transporters. The two closest homologs of NRT1.3, NRT1.4, and NRT1.1 share 51 and 47% amino acid sequence identities with NRT1.3, respectively. NRT1.1 is a dual-affinity transporter involved in both low- and high-affinity nitrate uptake, depending on cellular nitrate concentrations (Wang et al., 1998; Liu et al., 1999), and also facilitates the uptake of auxin in the absence of nitrate (Krouk et al., 2010). In contrast, NRT1.4 has been shown to be a low-affinity nitrate transporter (Chiu et al., 2004). Some other members are known to transport small organic anions as well as nitrate. NRT1.2 transports nitrate and abscisic acid (Huang et al., 1999; Kanno et al., 2012). NRT1.7, NRT1.9, and NRT1.10 (GTR2) play a role in the uptake of both nitrate and glucosinolates (Fan et al., 2009; Nour-Eldin et al., 2012). Although NRT1.3 remains to be characterized biochemically, its putative ortholog in *Medicago truncatula*, MtNRT1.3, has been shown to be a dual affinity transporter and also an ABA transporter (Morère-Le Paven et al., 2011; Pellizzaro et al., 2014). Given the cationic nature of polyamines, it is unlikely that NRT1.3 has the ability to transport polyamines, although its possibility cannot be excluded completely.

Nitrate is taken up from soil by the root tissue but its assimilation can take place in both the root and the shoot. *NRT1.1* and *NRT1.2* are involved in the uptake of nitrate from soil (Tsay et al., 1993; Wang et al., 1998; Huang et al., 1999; Liu et al., 1999). While *NRT1.5* is expressed in root pericycle cells close to xylem and acts in loading nitrate into xylem vessels for root-to-shoot transport (Lin et al., 2008; Chen et al., 2012), *NRT1.8*, which is expressed in root xylem parenchyma cells, and *NRT1.9*, which is expressed in companion cells of the root phloem, are also involved in regulating root-to-shoot nitrate translocation (Li et al., 2010; Wang and Tsay, 2011). *NRT1.7* is expressed in the phloem of the leaf vein, indicating that nitrate can be remobilized from older leaves to younger leaves (Fan et al., 2009). *NRT1.4* is expressed in the leaf petiole and may be responsible for nitrate homeostasis in leaves (Chiu et al., 2004). *NRT1.6* has been shown to function in delivering nitrate to the developing embryo in the seed (Almagro et al., 2008). We confirmed here that *NRT1.3* is highly expressed in flowers, stems, and leaves. Our results of *GUS* expression (**Figure 5**) suggest that, complementarily to other members of the NRT1/PTR family, *NRT1.3* may be expressed in mesophyll cells of the leaf and the cortex of the stem. However, because all *sper3* alleles show wild-type phenotype under normal growth condition (**Figure 1A**), other nitrate transporters may function redundantly in these parenchymal tissues. Interestingly, the *NRT1.3* promoter-*GUS* expression was not detected in epidermal cells. This might be related to the lack of chloroplasts in these cells. In shoot organs, nitrate assimilation is generally coupled to photosynthetic electron flow because the process of nitrite reduction occurs in the chloroplast stroma (Foyer and Noctor, 2002). The induction of *NRT1.3* expression by NO_3_^–^ is detected only in shoots (Okamoto et al., 2003). Furthermore, *NRT1.3* expression is up-regulated by light while *NRT1.1* and *NRT1.5* by sugar (Lejay et al., 2008). Taken together, as a member of the NRT1/PTR family, *NRT1.3* may play a role in the final step of nitrate supply to photosynthesizing cells. The strong fluorescence of NRT1.3-GFP at the cell periphery (**Figure 5F**) suggests that NRT1.3 is localized to the plasma membrane. However, its localization to endomembranes might also be possible. NRT2.7, one of the seven members of the NRT2 family, has been shown to be localized to the vacuolar membrane in seeds (Chopin et al., 2007).

We detected a reduced uptake of norspermidine in *sper3-1* (**Figure 6B**). This raises a possibility that polyamine transport is associated or at least coincided with nitrate transport in parenchymal tissues and its defect may cause the reduced uptake of polyamines. *sper3* mutants might have higher accumulation of nitrate in the root tissue and/or in the apoplast of shoot organs which could interfere with polyamine uptake. A recent study has revealed that mutants of a nitrate eﬄux channel, SLAH3, were more sensitive to ammonium, suggesting that the eﬄux of nitrate by SLAH3 alleviates ammonium toxicity in roots (Zheng et al., 2015). Although the sensitivity to polyamines in these mutants remains to be examined, SLAH3 might also serve to alleviate polyamine toxicity. On the other hand, considering that the initial uptake efficiency of norspermidine in *sper3-1* was not different from that in the wild type (**Figure 6B**), it is also possible that the incorporated polyamines can be metabolized more rapidly under reduced availability of nitrate in shoot parenchymal tissues of *sper3*.

According to the crystal structure of NRT1.1, it forms a homodimer in the inward-facing conformation (Parker and Newstead, 2014; Sun et al., 2014). If this is also the case with NRT1.3, the semi-dominant trait of *sper3-1* and *sper3-2* might be due to a dominant negative effect by the presumed dimerization with the wild-type protein rather than a haplo-insufficiency. The NRT1/PTR family protein is also known as a proton-coupled transporter. In NRT1.1, H356 in the 7th transmembrane domain (TM7) is a binding site of nitrate, while the ExxER motif in TM1 plays a role in proton binding and co-transport (Steiner et al., 1995). This motif interacts with K164-E476 salt bridge involved in nitrate uptake (Doki et al., 2013; Parker and Newstead, 2014). The E46K amino acid substitution in *sper3-1* occurs in the second E of the ExxER motif (**Figure 3C**), confirming the importance of this amino acid. Based on the phenotype of *sper3-1* and *sper3-2*, the A433V amino acid substitution in *sper3-2* may affect the function of NRT1.3 in a similar way to *sper3-1*. These alleles will provide useful tools for further biochemical study of this nitrate transporter.

## Conclusion

This study suggests that nitrate transport in the parenchymal tissue of the shoot is closely interrelated with polyamine uptake or metabolism. Although mutants of *NRT1.1*, *NRT1.2*, and *NRT1.4* showed normal sensitivity to spermine, involvement of some additional members in this interrelation may be possible, given the presence of multiple members of the *NRT1/PTR* family with various expression patterns. As a future study, it would be interesting to generate double and multiple mutants of these nitrate transporters and examine their sensitivity to polyamines.

## Author Contributions

WT, AI, HM, and TT conceived and designed the experiments. WT, AI, RT, and TT performed the experiments. WT, HM, RT, and TT analyzed the data. SS, KY, MY, MH, and SS contributed materials and analysis tools. WT and TT wrote the paper.

## Conflict of Interest Statement

The authors declare that the research was conducted in the absence of any commercial or financial relationships that could be construed as a potential conflict of interest.

## Footnotes

1 http://www.arabidopsis.org
