# Supplementary material for: Polyamine Resistance Is Increased by Mutations in a Nitrate Transporter Gene NRT1.3 (AtNPF6.4) in Arabidopsis thaliana
Source: Front Plant Sci. 2016 Jun 13;7:834. doi: 10.3389/fpls.2016.00834 (PMC4904021; doi:10.3389/fpls.2016.00834)
Supplement: Supplementary file 1 [file Image_1.PDF]

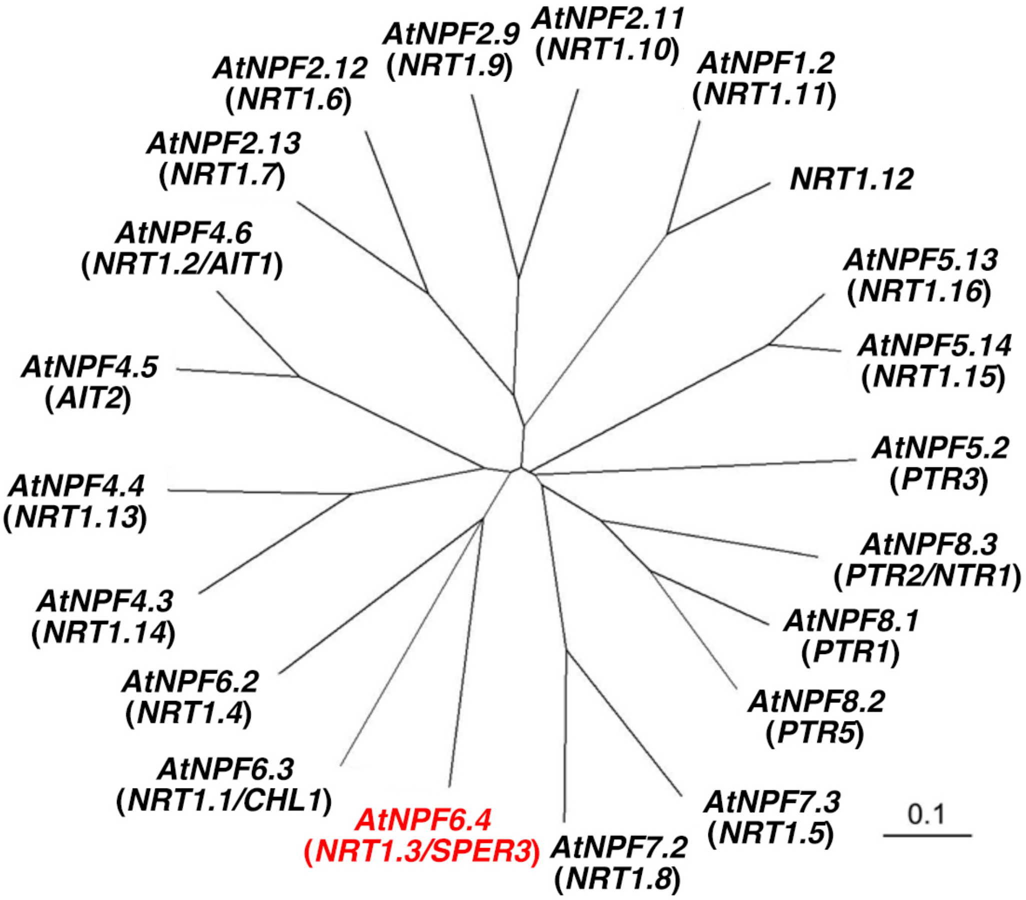

Fig. S1

Figure S1. Unrooted molecular phylogenetic tree of the *NRT1* family members from *Arabidopsis thaliana*. The tree was constructed by Clustal W analysis (DDBJ; http://clustalw.ddbj.nig.ac.jp/) using the neighbour joining algorithm. Bar represents 0.1 amino acid substitutions per site.

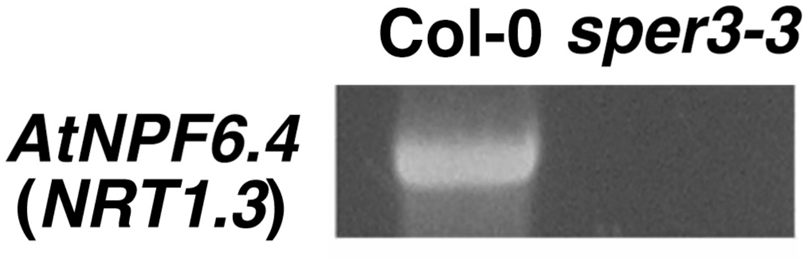

Fig. S2

Figure S2. Detection of the *NRT1.3* mRNA in wild-type and *sper3-3* seedlings. Total RNA was prepared according to the SDS-phenol method and reverse-transcribed with oligo-dT primers and PrimeScript^{TM} reverse transcriptase (Takara, Kyoto, Japan) at 42°C for 1 h. The resulting first-strand cDNA was used for a 40-cycle PCR amplification of 94°C for 30 s, 55°C for 30 s, and 72°C for 2 min with *NRT1.3*-specific primers, F, 5'-CTGTC ACAGC TCTGG GAGTG-3' and R, 5'-AGCAA TCACG GCGAA GAGAG-3'.

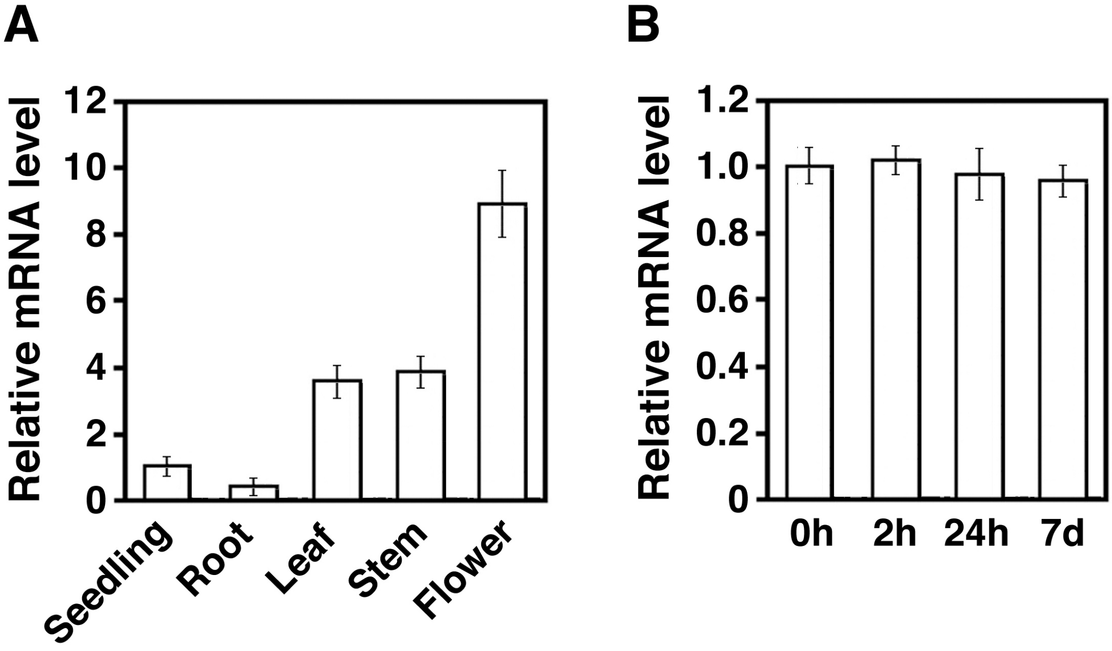

Fig. S3

Figure S3. RT-PCR analysis of *NRT1.3*. (A) Relative mRNA levels of *NRT1.3* in wild-type seedlings, roots, leaves, stems, and flowers. **(B)** Relative mRNA levels of *NRT1.3* in wild-type seedlings *treated with* 100 µM spermine. Total RNA preparation, cDNA synthesis, and *NRT1.3*-specific PCR primers are as described in Fig. S2 legend. PCR reactions were performed using KAPA SYBR FAST qPCR Kit (KAPA Biosystems) and the DNA Engine Opticon2 System *(Bio-Rad*). *ACTIN8* (At1g49240) *was used* as an internal standard in the reactions (Tong et al., 2014). *Data from three independent biological replicates each* with two technical replicates are expressed as means ± SE.

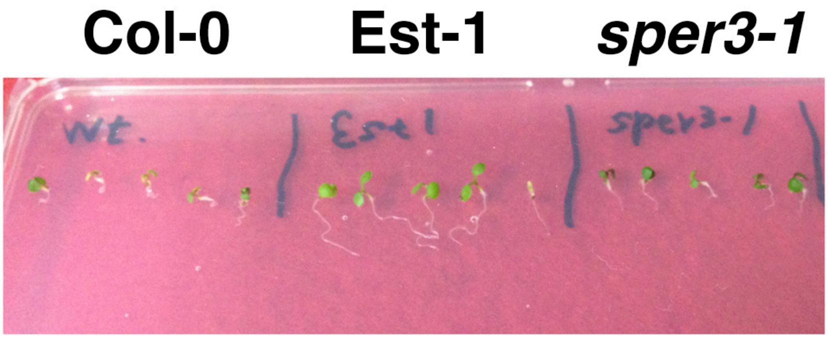

Fig. S4

Figure S4. Paraquat sensitivity of the wild-type accessions of Col-0 and Est-1, and *sper3-1* in the Col-0 background. Three-day-old seedlings grown with 100 nM paraquat are shown.
