# Supplementary material for: Polyamine Resistance Is Increased by Mutations in a Nitrate Transporter Gene NRT1.3 (AtNPF6.4) in Arabidopsis thaliana
Source: Front Plant Sci. 2016 Jun 13;7:834. doi: 10.3389/fpls.2016.00834 (PMC4904021; doi:10.3389/fpls.2016.00834)
Supplement: Supplementary file 2 [file Table_1.PDF]

Table S1. List of PCR primers used for mapping and genotyping.

Chromosome position (kb)	Primer name	Primer sequence	Enzyme for CAPS	Fragment length (bp)
III-6495	MCB22-F	5'-TTATCGGATGTCCCTGAAGA-3'	*Cla*I	Col-0: 510, 300
	MCB22-R	5'-TTTCGAACAATTCACGCGCT-3'		L*er*: 810
III-7508	MHC9-F	5'-GTCTTTGAAGAGAAACAGAGGA-3'	*Nsp*V	Col-0: 510, 150
	MHC9-R	5'-AAGACGCCATGATTCTGTTGAT-3'		L*er*: 660
III-7609	MIL23-F	5'-CTTCTTGTAGTCTAGTGGTC-3'	*Sac*I	Col-0: 510, 300
	MIL23-R	5'-CTGAATTGGGCTGCAGGTTA-3'		L*er*: 810
III-7713	MZN24-F	5'-CCGAACCGAAATCAACTGTACC-3'	(SSLP)	Col-0: 1220
	MZN24-R	5'-CTGAACGAGAGGAACATGGAGT-3'		L*er*: 505
III-8120	F5N5-F	5'-TTTGATGGCTGCGATAATGCCAC-3'	(SSLP)	Col-0: 2200
	F5N5-R	5'-TTGATTAGTGGATCCGCAAACAA-3'		L*er*: 340
III-8336	M255-F	5'-AGGGAAGTGGATTGGCTCCA-3'	*Scr*FI	Col-0: 510, 500
	M255-R	5'-GTGTTTAGTAATGAATAATCATC-3'		L*er*: 1010
III-9100	MJL12-F	5'-GATGGGAGGCTAGAGACTCATA-3'	(SSLP)	Col-0: 650
	MJL12-R	5'-CGACTGAGAGATTCAGAAACCC-3'		L*er*: 490
III-7628.5	sper3-1F	5'-CTTAATGTGAAGGGAGCGAGCTCTCA-3'	*Dde*I	Col-0: 200, 25
	sper3-1R	5'-AGCTGTGACAGAAGTTGAGATTGC-3'		*sper3-1*: 225
III-7627.5	sper3-2F	5'-TCTATTTCTCCATCAGCGTC-3'	*Sac*II	Col-0: 710, 250
	sper3-2R	5'-CAGCTTTTGTCTGTAACCCT-3'		*sper3-2*: 960, *sper3-3*:-
-	pBI-LB	5'-AACCAGCGTGGACCGCTTGCTG-3'		*sper3-3*: 710 (2F+LB)
